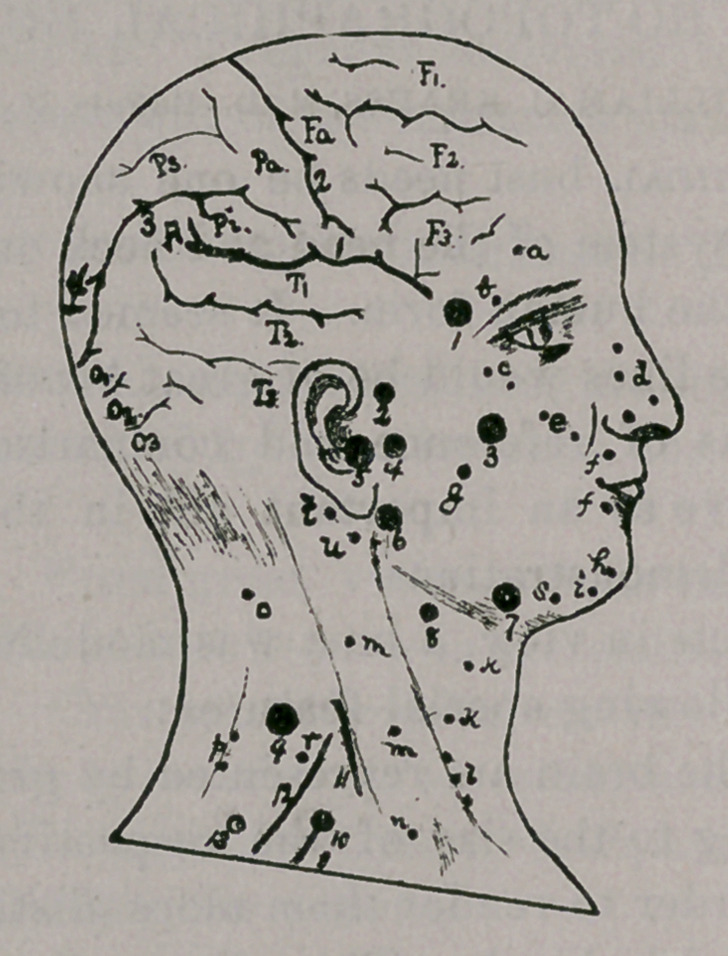# A Neuro-Topographical Bust1Exhibited at the meeting of the American Neurological Society, Washington, D. C., September 22, 23, and 24, 1891.

**Published:** 1892-03

**Authors:** William C. Krauss

**Affiliations:** Buffalo, N. Y.


### Selections.

---

### A NEURO-TOPOGRAPHICAL BUST.[1]

By WILLIAM C. KRAUSS, M. D., Buffalo, N. Y.

A NEURO-TOPOGRAPHICAL bust needs be one showing the topography of the nervous system of the head and neck on a model closely resembling that of the human form. It seemed to me that a bust constructed on these lines would be of great benefit to the neurospecialist as a means of reference and comparison, while to the teacher it would serve as an important aid in the elucidation of facts in class-room demonstrations.

With these objects in view, a bust was modelled in plaster, life size, offering the following special features:

The fissures of the brain are represented by grooved lines, deep or shallow, according to the size of the respective fissures in the human brain. In order to render them more distinct and visible, they have been traced in black. These lines are not intended to represent accurately the intricate windings of the various fissures, but only the general outlines and the relations of the various fissures to one another.

The position of the several centers presiding over the functions of the body, as far as known, could be easily designated on the bust, thus keeping before our eyes the brilliant achievements constantly being made in this branch of our science.

The face and neck represent the various electro-motor points of the muscles and nerves as determined by Erb, v. Ziemssen, *et al.* The motor points of the trunk and various branches of the trigeminus, the hypoglossal, accessorius, etc., are indicated by circles slightly raised, which, in order to make them more apparent, have been painted yellow. The course of the phrenic nerves, and the several trunks of the brachial plexus in the neck are marked by slightly grooved lines, also painted yellow.

1. Exhibited at the meeting of the American Neurological Society, Washington, D. C., September 22, 23, and 24, 1891.

In contrast to the nerves, the motor points of the muscles are represented by smaller circles, which have been painted red.

The designation of the various points has been purposely omitted on the bust, but may easily be done, either by small printed slips, or by figures and a reference table.

The work of the moulder, Mr. Gustave Freret, of 155 East 50th street, New York, has been skilfully and well done, and he is prepared to furnish duplicates at any time.

### EXPLANATION OF PLATE.

### CRANIAL REGION.

1. Sylvian fissure. 2. Central fissure (Rolando). 3. Parietal fissure. 4. Occipital fissure. F1. Superior frontal convolution (superfrontal). F2. Middle frontal convolution (medi-frontal). F3. Inferior frontal convolution (subfrontal). Fa. Ascending frontal convolution (precentral). Pa. Ascending parietal convolution (postcentral). Ps. Superior parietal convolution (parietal). Pi. Inferior parietal convolution (subparietal). A. Angular convolution. T1. Superior temporal convolution (supertemporal). T2. Middle temporal convolution (meditemporal). T3. Inferior temporal convolution (subtemporal). O1. Superior occipital convolution. O2. Middle occipital convolution. O3. Inferior occipital convolution.

### FACIAL REGION.

1. Trifacial nerve; superior branch. 2. Trifacial nerve; superior branch. 3. Trifacial nerve; middle branch. 4. Trifacial nerve; middle branch. 5. Trifacial nerve; trunk. 6. Trifacial nerve; inferior branch. 7. Trifacial nerve; inferior branch. 8. Hypoglossal nerve. 9. Accessorious nerve. 10. Erb's point (supraclavicular point). 11. Phrenic nerve. 12. Brachial plexus. 13. Axillary nerve.

### MUSCLES.

a. Frontalis. b. Corrugator supercilii. c. Orbicularis palpebrarum. d. Nasal muscles. e. Zygomatic muscles. f. Orbicularis oris. g. Masseter. h. Levater menti. i. Quadratus menti (depressor labii inferioris). k. Platysma myoides. l. Hyoid muscles. m Sterno-cleido-mastoid. n. Omo-hyoid. o. Splenicus. p. Trapezius. r. Levator anguli scapuli. s. Triangularis menti (depressor anguli oris). t. Stylo hyoid. u. Digastric.

—*Journal of Nervous and Mental Diseases.*

## Figures and Tables

**Figure f1:**